# Social life cycle sustainability assessment of dried tomato products based on material and process selection through multi‐criteria decision making

**DOI:** 10.1002/jsfa.13974

**Published:** 2024-10-26

**Authors:** Dilber Ayhan, Francisco Astorga Mendoza, Muhammed Rasim Gul, Izzet Ari, Hami Alpas, Mecit Halil Oztop

**Affiliations:** ^1^ Scientific and Technological Research Council of Türkiye (TUBITAK) Ankara Türkiye; ^2^ Lomartov Applied Innovation Engineering Valencia Spain; ^3^ Food Engineering Middle East Technical University Ankara Türkiye; ^4^ Graduate School of Social Sciences Social Sciences University of Ankara Ankara Türkiye; ^5^ Food Engineering, Earth System Science Middle East Technical University Ankara Türkiye

**Keywords:** social life cycle assessment, product life cycle, tomato products, Mediterranean products, multi‐criteria assessment, integrated assessment

## Abstract

**BACKGROUND:**

Tomatoes are a significant product of the Mediterranean region and a crucial component of the Mediterranean diet. The formulation of dried tomato products enriched with proteins and bioactive compounds could be a strategic approach to promote adherence to the Mediterranean diet. Six different novel tomato products were analyzed using different protein enrichment sources (pea proteins and leaf proteins) and drying technologies (hot‐air dryer, microwave vacuum dryer, and conventional dryer). The novelty of this approach lies in combining product‐specific criteria with global societal factors across their life cycles. Using 21 criteria and an analytic hierarchy process (AHP) survey of experts, the social sustainability score for each product was determined through a multi‐criteria assessment.

**RESULTS:**

The tomato product's life cycles have minimal regional impacts on unemployment, access to drinking water, sanitation, or excessive working hours. However, they affect discrimination, migrant labor, children's education, and access to hospital beds significantly. The study identified nutritional quality as the top criterion, with the most sustainable design being a tomato bar enriched with pea protein and processed using microwave vacuum drying.

**CONCLUSION:**

The study revealed that integrating sensory and nutrient compounds into social sustainability assessments improves food sustainability and provides a practical roadmap for social life cycle assessments of food products. It emphasized the importance of considering global social issues when reformulating Mediterranean products to ensure long‐term adherence to the Mediterranean diet. Incorporating social factors into sustainability scores can also enhance the effectiveness of product information for conscious customers. © 2024 The Author(s). *Journal of the Science of Food and Agriculture* published by John Wiley & Sons Ltd on behalf of Society of Chemical Industry.

## INTRODUCTION

The Mediterranean diet is globally recognized for its health benefits, with tomato as one of its key components. Tomatoes are rich in essential nutrients and abundant in antioxidants like lycopene, known for its potential to reduce the risk of cancer and cardiovascular diseases.^1^ Tomato has very healthy components such as dietary fibers, lycopene, *β*‐carotene, proteins, vitamin C, phenolics, and flavonoids.^2^ Tomato processing is a very large industry, with 40 million tons of tomatoes processed annually.^3^

There is a concerning decline in adherence to the Mediterranean diet, particularly among younger generations. To reverse this trend, researchers and food scientists are exploring innovative ways to reformulate traditional Mediterranean products, enhancing their appeal and nutritional value.^4^

One such avenue is the development of tomato‐based products enriched with proteins and bioactive compounds. These reformulated products aim not only to preserve the cultural significance of tomatoes in the Mediterranean diet but also to enhance their nutritional profile, potentially revitalizing interest and adherence to this traditional dietary pattern. Some of these novel products include tomato leathers, a healthier alternative to traditional fruit leathers, made with minimal ingredients like tomato juice, olive powder, pea protein isolate, and salt.^1^ Tomato snack bars have also been developed, incorporating ingredients such as olive powder, pea protein isolate, tomato powder, and low‐methoxylated pectin.^4^

**Figure 1:**
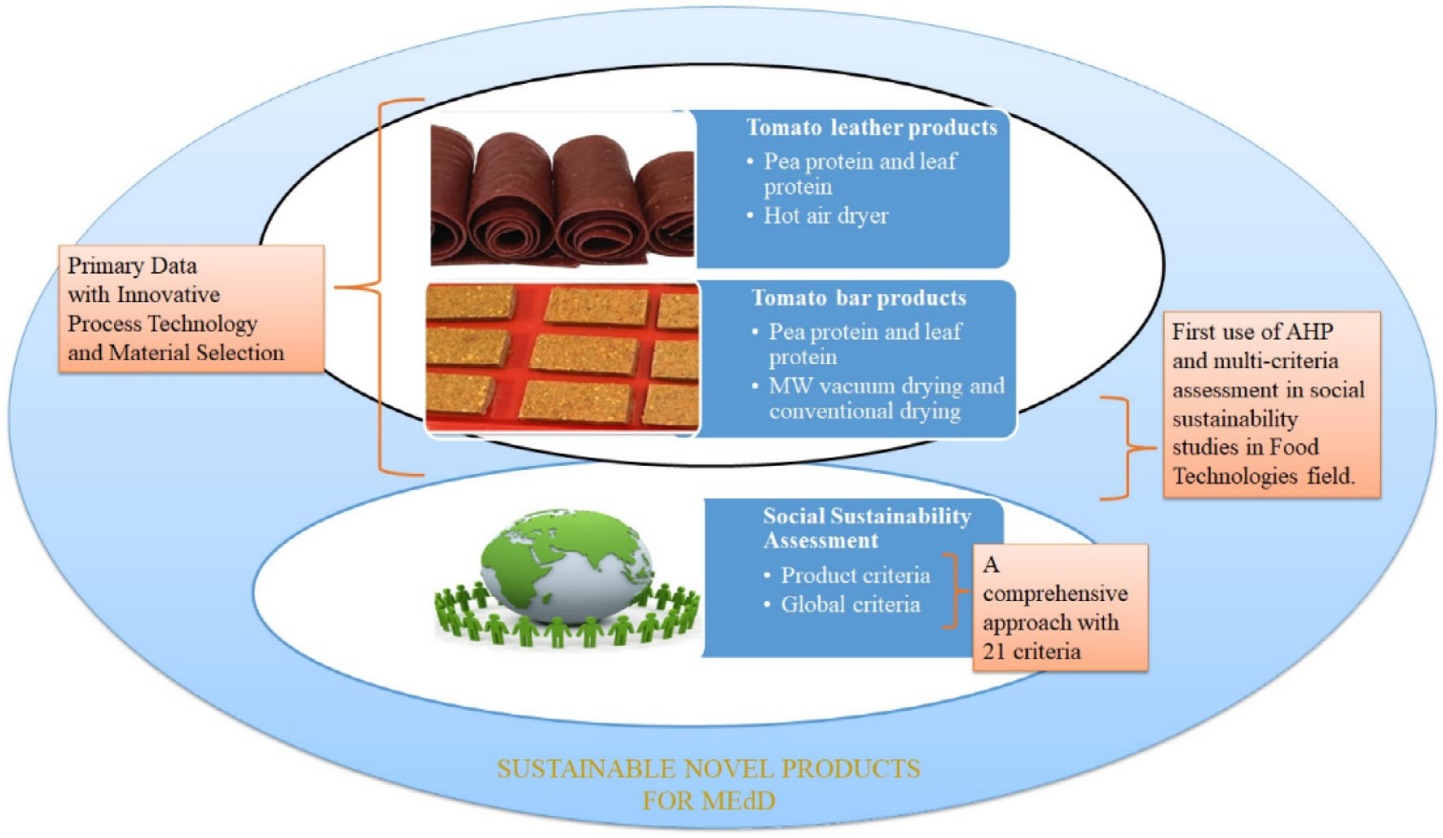
Illustration of main proposal.

Studies have explored various processing techniques, including microwave‐vacuum drying and conventional hot air drying, to retain the nutritional and sensory qualities of these products. The formulations of these tomato‐based products aim not only to enhance their appeal but also to fortify them with proteins and bioactive compounds, aligning with the goals of promoting adherence to the Mediterranean diet and improving overall health outcomes. As Fig. 1 shows, although innovative formulations and processing techniques hold promise for enhancing the nutritional and sensory aspects of tomato products by considering product utility (positive impact), a comprehensive assessment of their social sustainability (with negative impacts) is essential to ensure their long‐term viability and alignment with the principles of the Mediterranean diet.

**Figure 2:**
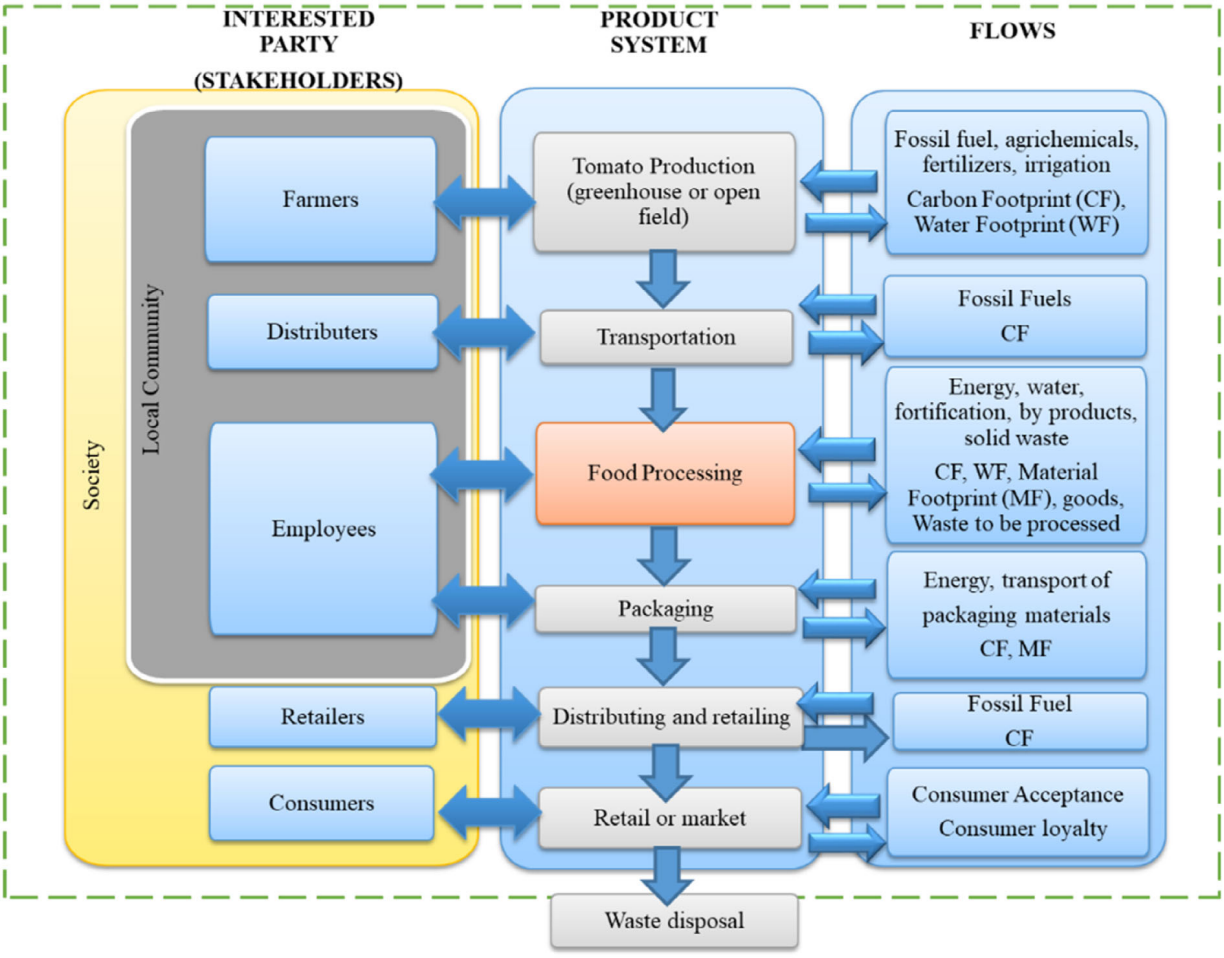
System boundary of the social LCA assessment of the dried tomato products.

Social impacts in food chains include negative effects like poor working conditions, local community health and safety issues, and productivity impacts, as well as positive effects like employment opportunities. Issues like forced labor, human rights violations, and inadequate wages are important.^5^ Growing consumer awareness and demand for socially responsible products, with a willingness to pay more for such options, is evident.^5^, ^6^ This trend is seen in sectors like the wine industry, where sustainability claims influence purchasing decisions. Companies are increasingly measuring and enhancing their social impacts to align financial goals with social well‐being.^7^, ^8^ In the food industry, this involves fair labor practices, ethical sourcing, and community engagement.^9^ Integrating social sustainability into business models can help companies create a more equitable food system and meet the demands of consumers who prioritize ethical and sustainable practices.^10^

The theoretical development of the environmental, economic, and social pillars of sustainability varies.^11^ Social life cycle assessment (LCA) applications are less mature than environmental LCA,^12^ with only 9% of studies focusing on social aspects in comparison with 40% focusing on environmental aspects.^13^ Research is ongoing due to challenges and developments.^14^, ^15^, ^16^

Social sustainability in food systems often centers on specific life cycle stages, like harvesting and cultivation.^17^, ^18^, ^19^ A recent review found only six papers on social LCA in agri‐food crops.^20^ Existing studies generally overlook social factors at a global level, such as workers' conditions and governance.^21^ In addition, the author identified twenty social LCA case studies that address up to ten criteria, which are limited for comprehensively measuring social sustainability.

This study addresses this gap by evaluating the social sustainability of dried tomato products across their entire life cycle. Unlike typical social sustainability studies, which focus on one or two criteria, this sustainability study of tomato food products spans seven impact subcategories with 21 criteria. Topics include nutrient content and health benefits, focusing on areas such as microwave drying,^22^ bioactive compound retention,^23^ green extraction methods,^24^ and plant protein sources.^25^ Other research covers a variety of social issues like welfare and job security in agriculture.^18^, ^26^, ^27^

Overall, there remains a limited understanding of the social impacts across the entire food chain, primarily due to the complexity of such analyses and the lack of interdisciplinary approaches.^28^ Addressing gaps in understanding of social impacts across the food chain, this study uses surveys and interviews to monitor cultural, product‐quality, and health aspects in purchasing practices and traceability. Context‐specific sustainability assessments^29^ guide this comprehensive approach to examine social sustainability of dried tomato products, informed by political consensus, stakeholder input, and the literature.^21^ Integrated criteria yield a social sustainability score, evaluating broader societal implications of food development.

Unlike environmental risks, social sustainability focuses on human health benefits throughout a product's life cycle.^30^ The challenge in the proposed sustainability evaluation involves balancing the system dynamics of benefits and risks belonging to the criteria, with broader supply chains increasing global impact precision. This research is pioneering in applying the ‘technique for order by preference by similarity to ideal solution’ (TOPSIS) and ‘simple additive weighting’ (SAW) methods to calculate social sustainability scores in the food technologies field.

The TOPSIS and SAW methods have been proven to have the best discriminative ability in comparative analysis between different alternatives.^31^ These methods were selected as the eight best methods among the 56 different multi‐criteria methods.^21^ Their scores are normalized and weighted differently. The TOPSIS method, which finds the best solution based on similarity to an ideal solution, was 52% effective among five multi‐criteria decision analysis (MCDA) techniques.^32^ Simple additive weighting, which uses weighted averages, had a significance of 0.09%. In principle, the two methods differ in the normalization scheme and in the way in which the performance score is calculated. Whereas TOPSIS and SAW have been explored in construction building technologies,^32^ the novel application introduced in this study extends the use of MCDA techniques to food sustainability. The different MCDA methods were compared and AHP emerged as the most widely used.^32^ The literature has not given give a sustainability calculation but recommended its use in food waste management. Monte Carlo simulation was utilized for carbon and water footprints in Italian red wine^33^ without calculating a sustainability score.

Thus, to solve the complex problem of processing 21 criteria into a quantifying score of sustainability, the current study uniquely applies TOPSIS, SAW, and AHP to the food product life cycle. Other MCDA methods, such as PROMETHEE and ELECTRE, were not chosen for evaluation because they provide dominance rankings among the proposed solutions rather than generating an index, which would not be as useful for this particular assessment.^34^

Sustainability assessments should be holistic and specific.^21^ Micro‐level studies require primary data, whereas macro‐level studies use databases.^12^ Product‐specific studies are more common than global impact studies.^13^ This research addresses the gap by combining product‐specific indicators with social global issues to design sustainable products in food technologies. It investigated the impact of different protein sources (pea and leaf protein) and drying technologies (tray, microwave vacuum, and conventional dryers) of novel products on social sustainability. It evaluated tomato food products using 21 criteria, including product‐specific and global level indicators, across a cradle‐to‐grave life cycle. This comprehensive approach provides a methodological roadmap for assessing new products' contributions to social sustainability.

## MATERIALS AND METHODS

### Materials

Sustainability evaluations were made on the six dried tomato products of the ongoing Horizon Project of Functionalized Tomato Products (FunTomP) project – namely, two different tomato leather products and four different tomato bar products, as defined in Table 1. Preliminary information on the raw materials and process activities was collected from the project design engineers. The tomato bar products (45 g) were produced by using conventional and microwave vacuum dryers after mixing the amount of ingredients^4^ given in Supporting Information, Table S1. These bars are rich in protein and are made using Roma‐type tomatoes. The olive powders used in the recipe are sourced from green olives from the Marmara region in Türkiye. The bars are made using pea and rubisco proteins and are available in a common bar shape. Depending on the type of protein used, the color of the bars varies. Bars with pea protein are reddish brown, whereas those with rubisco protein are dark green. The texture of the bars is crunchy and gritty.

**Table 1:** Description of the dried tomato products

Products (A refers Alternative, representing product)		Product features
Tomato leather product‐ 1 (R_Tray_leather)	A1	Tray dryer (Tray) was used
		Enriched with Rubisco protein
Tomato leather product‐ 2 (PP_Tray_leather)	A2	Tray dryer was used
		Enriched with pea protein (PP)
Tomato bar product‐1 (R_MW_Bar)	A3	MW vacuum dryer (MW) was used
		Enriched with Rubisco protein
Tomato bar product‐ 2 (PP_MW_Bar)	A4	MW vacuum dryer is used
		Enriched with pea protein
Tomato bar product‐ 3 (R_Conv_Bar)	A5	Conventional air dryer (Conv) is used
		Enriched with Rubisco protein
Tomato Bar Product‐ 4 (PP_Conv_Bar)	A6	Conventional air dryer is used
		Enriched with pea protein

Fresh tomatoes were selected, processed (85 °C, 3 min) by a thermal mixer, and incorporated into a snack bar matrix by mixing with pectin, protein, tomato powder, salt, and spices. Ingredients were mixed using high‐shear homogenization, and snack bars were molded and kept in the fridge for a day. A microwave‐vacuum dryer was used for drying at 60% power (maximum power: 2 kW), 0.5 atmosphere vacuum pressure for 10 min. The total weight of the tomato snack bars decreased from 128 to 48 g by drying. A conventional oven applies hot air drying at 120 °C for 90 min. Drying parameters were selected based on tomato snack bars' water activity (~0.6).

The leather products were produced by mixing and homogenizing the ingredients of tomato juice, protein source, salt and olive powder. Unlike the bar products, the leather products were dried by a tray dryer. The primary component of the leather was tomato juice, which had an average Brix of 5.2 and a pH of 4.2. Olive powder, pea protein isolate, and salt were added to this, each at a concentration of 0.5% (w/w). The mixture was first pre‐homogenized and subsequently, a high‐pressure homogenizer (GEA Group, Düsseldorf, German) was employed to process the mixture through two passes at 500 bars to ensure thorough integration of the pea protein into the matrix.^1^

For all the tomato products, two different protein sources, pea protein and Rubisco protein extracted by a previously described method,^35^ were used to enrich the products.

Fresh tomato (*Solanum lycopersicum*) and tomato pomace were obtained from Kraft Heinz (Balıkesir, Türkiye). Pea protein isolates were obtained from Vegrano (Istanbul, Türkiye). Olive powder was prepared according to a procedure described in the literature.^36^ Mint and salt were purchased from a local market (Ankara, Türkiye). All of the chemical components were obtained from Merck (Darmstadt, Germany).

The ‘Social Hotspot 2019 Subcat & Cat Method w Damages/Equalsubcatweights’ method^37^ within the Social Hotspots Database (SHDB) was used to reach the global level social inventory. There are two main types of social pathway.^38^ The first uses reference point scales to estimate social risks based on performance levels, with thresholds set by standards and best practices. The second type employs cause‐and‐effect chains using three methods: identifying new indicators, validating existing variables through experiments, and applying known models. The SHDB provides social pathways based on reference scales, analyzing inventory data across sectors and regions^39^ with subcategories given in Supporting Information, Table S2.

The SHDB can be applied in sector‐specific industries.^40^ It has been utilized to assess sugarcane production's social sustainability.^41^ The SHDB requires data to be input in 2011 USD, so 2023 prices are adjusted to 2011 values by applying a deflator of 1.39.^42^ Electricity during the processes is priced at 3.46 TRY kWh^−1^ for industrial use.^43^ The calculations are based on an exchange rate of 30 TRY per dollar as of 10 January 2024.

The inventory tables for each product were created in Excel. SimaPro by the company PRé Sustainability (Amersfoort, Netherlands) was used to obtain the social impact data from the SHDB. The AHP and MCDA calculations were performed in Excel.

### Social life cycle analysis

Social corporate responsibility evolved throughout the 19th and 20th centuries,^44^ eventually transforming into social life cycle assessment (SLCA) guidelines developed by Society of Environmental Toxicology and Chemisty (SETAC) and UN working groups.^38^ Standardized life cycle assessment methodology^45^ employs the steps of goal and scope definitions, inventory analysis, and impact assessment. We then use TOPSIS and SAW to consolidate and integrate the LCA results for a sustainability score. The primary data from the ongoing FunTomP project was used for the product‐specific individual criteria such as nutrition and sensory analysis. Material flow analysis was conducted during LCA by using the inventory tables constructed by primary data. Secondary data from SHDB was used for global level criteria, focussing on issues like child labor, employment, and human rights.

#### Step 1: goal and scope definition

The goal of this analysis was to determine the social sustainability performance of the novel and dried tomato products. The target audience for the results of the social performance of the products was project members who design the products and adjust the system requirements to achieve better sustainability. Conscious consumers also represent a key target group for promoting more sustainable food products in their diets. During the calculations, the functional units of the system were 45 g for the tomato snack bar and 17 g for the tomato leather, considering the quantity of final product ready for packaging. Figure 2 shows the system boundary for the social sustainability assessment of dried tomato products with stakeholders and multiple stages along the food value chain.

The system boundary extends from primary production to the end user, the consumer. The boundary is drawn only by ignoring waste disposal in households. Thus, it has a cradle‐to‐market approach. If the aim was to compare only the drying technology, a gate‐to‐gate approach could be applied, where the system boundary would only include food production.

#### Step 2: life cycle inventory

The social inventory was collected for each product. Information on the energy used, costs of inputs, recycling, and allocations were calculated. In addition to materials, inventories for processes were also considered. The transportation of raw materials (from farms or warehouses to the plant) and final products (from the plant to retail) was calculated using transportation data and distance estimates from the SHDB.^46^, ^47^ For the transport inventory, freight was given by trucks (7.5–16 metric tons). For 10 tons of trucks traveling at 90 km/h, 0.287 L of fuel per kilometer were reported.^48^ The transport distance was assumed to be 75 km from farm to factory, which is double the distance of a truck traveling from the factory and back. For processed products, a distance of approximately 500 km from the factory to the retailer was assumed.^46^, ^47^ The preferred packaging material was from biodegradable polylactic acid, which was extracted from sugar beet for use in packaging.^46^, ^47^ When the output of one process was a material input to another process, food allocations were considered during the calculations. For example, 10% waste of fresh tomatoes from tomato juice production was allocated to tomato powder production; 25% percent of sugar beet was allocated to sugar beet leaves.^49^ All waste was used during production, so none was taken to the waste management unit.

The impact increased and decreased according to the functional unit. However, in terms of micro‐level social assessment, the product‐specific response may not be proportional. For example, the benefits to research and innovation capacity and sensory analysis criteria are product‐specific and cannot be functionalized. For each step in the food supply chain, the origin of the entries for each product under comparison should be specified in terms of sector and region^44^ as shown in Supporting Information, Table S5.

#### Step 3: social sustainability assessment

It is recommended that the criteria that are significant for stakeholders should be selected and integrated into the product/process system and that perspectives should be provided on causes and impacts.^50^ In this stage, a suitable set of social criteria was identified from the literature recommendations and the preferences of the expert group of the AHP survey. The predetermined set of criteria was combined with the sustainability tools and indicators for the food supply chain^28^, ^51^, ^52^ and indicators of sustainable development goals.^53^, ^54^ The key literature on social life cycle databases^30^, ^40^ and sustainability reporting guidelines^10^, ^29^ was adopted.

The relevance of the criteria was first tested by the least mean squares method. When the performance of the products is similar, the criterion is dropped from the model, even though it might be considered important. For example, water content was similar in each product, which makes no difference when using it as a decision‐making parameter. Social criteria, for which data were not available through primary or secondary sources, were also discarded in the model. Three impact categories, seven impact subcategories and 21 criteria (attributes) were determined to calculate the social sustainability of the tomato products according to ISO 14044:2006 and the key references listed in Table 2.

**Table 2:** Social impact categories, subcategories and criteria

Impact category	Impact subcategory	Category criteria	Key references
Workers/employers (IC1)	Employment (SC1)	Unemployment (C1)	38, 55, 56, 57
		Discrimination (C2)	
		Migrant Labor (C3)	
		Child labor (C4)	
	Labor rights (SC2)	Forced labor (C5)	32, 58, 59, 60
		Excessive working time (C6)	
		Freedom of association (C7)	
		Labor laws (C8)	
Consumers (IC2)	Product utility for sensory satisfaction (SC3)	Sensory analysis (C9)	32
	Product utility for nutritional quality (SC4)	Total phenolic compound and flavonoid content (C10)	24, 25, 52, 61, 62, 63
		DPPH antioxidant activity (C11)	
		Protein content (C12)	
Society (IC3)	Benefits to research and innovation (SC5)	Research and innovation capacity (C13)	29, 51
	Infrastructural improvements (SC6)	Access to drinking water (C14)	58
		Access to sanitation (C15)	
		Children out of school (C16)	
		Access to hospital beds (C17)	
	Human rights (SC7)	Gender equity (C18),	58
		Indigenous rights (C19),	
		High conflicts (C20),	
		Corruption (C21)	

The reasons for selecting macro‐level impact criteria from the SHDB in this study are explained below for each stakeholder in the food value chain.

##### Employment

This category of impact is highly relevant in the Guide to Social Life Cycle Assessment.^38^ The Indicateurs de Durabilité des Exploitations Agricoles (IDEA) approach assesses social sustainability through contributions to employment.^64^ It is used as an agricultural employment criterion.^65^ Discriminatory employment is also used as an indicator of social assessment.^66^ Unemployment, migrant labor, and child labor are themes under the impact category of ‘Workers’ rights and decent work’.^58^ The sustainable rice platform (SRP) methodology also proposes child labor as an indicator of social sustainability. As a result of the previous literature, unemployment, discrimination, gender equality, child labor, and migrant labor were all aggregated under the employment category in this social assessment model.

##### Labor rights

The social LCA model^58^ has already incorporated ‘labor rights and decent work’. Labor rights have also been studied^64^ within the holistic social assessment model, focusing on work hours.^67^ In this study, forced labor, excessive working hours, participation in freedom of association, and labor laws were aggregated under the labor rights impact category.

##### Infrastructural improvements

This impact subcategory aims to embed infrastructure arising from product supply chains into the social model. ‘Access to drinking water, access to sanitation, children out of school, and access to hospital beds’ are included in the social LCA model.^39^ ‘Social infrastructures and services’ criterion also quantifies infrastructure improvements in the evaluation of agricultural systems.^65^

##### Human rights

This category is organized according to the themes of gender equality, indigenous rights, high conflict, and corruption already presented by the SHDB.^39^ The risk of corruption negatively affects all social groups and prevents the risk to human rights from increasing.^68^ Equity is also considered within the social concept of sustainability^69^ and gender equity in the social viability of irrigated agriculture systems.^67^

In the constructed social assessment model, the consumer category is based solely on product quality criteria. The Indicateurs de Durabilité des Exploitations Agricoles (IDEA) method also uses product quality criteria within a social sustainability approach.^64^ Its characterization focuses on product utility, encompassing both sensory and nutritional analysis. The product utility criterion can be defined as the perception of the consumer as well as the functionality of the product.^29^

Sensory analysis was performed and scored by Seluz Fragrance and Flavor Company (Istanbul, Türkiye) within the FunTomP project. The samples intended for evaluation were stored at 21°C in a dark environment for 3 days. They were presented to the panelists in identical sizes and equal portions. Each product was coded with a three‐digit random number and assessed by a team of three trained panelists. The evaluation process consisted of three stages. During the first session, the panelists assessed the products individually. In the second session, the individual evaluations were discussed openly among all panelists, reaching a consensus on the intensity of the taste characteristics. In the final session, suggestions for enhancing the flavor perception of each product were explored.

Nutritional content was also included as an indicator of product benefits. Antioxidant capacity protects the human body from cancer when adequately obtained from dietary sources, including carotenoids, ascorbic acid, and polyphenols.^70^ The DPPH antioxidant capacity was assessed based on the finding from the work packages of FunTomP Project. The phenolic content varied depending on the drying process, as temperature and time are important for degradation after adding olive powder as a raw material. The protein content was also increased due to the added protein. As the products are dry, food safety does not seem to be important in this social sustainability model, although it was previously added at the farm level.^69^

Research and development expenditures and improvements are considered as a social criteria of sustainability,^51^ as a socioeconomic criteria^71^ and as a ‘technology development’ factor in the Guideline for Social Life Cycle Assessment.^29^ Under the community stakeholder category, a product‐specific criterion of ‘benefit to research and innovation’ was added to the sustainability model to contribute to society. It was characterized by the number of research and innovation outputs of each product such as articles, patents, papers, posters, and dissemination activities achieved in the FunTomP project. In summary, the product specific parameters of the six tomato products are provided in Table 3.

**Table 3:** Product‐specific parameters of the social dimension

Products (A1–A6)	Sensory score (out of 5.00)	Sum of TPC and flavonoids (after normalization)	DPPH (EC_50_ mg ml^‐1^)	Protein content (%)	Research and innovation outputs
A1	2.75	0.144	1.431	0.493	20
A2	3.50	0.169	1.803	0.493	14
A3	2.20	0.489	0.460	0.781	19
A4	2.80	0.326	0.670	7.813	16
A5	2.75	0.487	0.600	0.781	19
A6	3.50	0.385	0.630	7.813	14

### Analytic hierarchy process for prioritization

The AHP is a widely acknowledged and valued method of MCDA due to its structured approach and consistency in decision‐making processes.^72^, ^73^ The experts' priorities for the impact categories and subcategories can be determined by creating a hierarchy of priorities.^74^, ^75^ This allows us to compare two alternatives and select the best one.^74^

To prioritize the impact categories and subcategories (dimensions), an AHP survey was conducted by interviewing 11 experts according to the relevant information in Supporting Information, Table S3. In fact, at least eight experts should be selected.^57^ The relative importance of the experts is assumed to be equal. The criteria are compared in pairs in Supporting Information, Table S4 on a scale^74^, ^75^ of 1 = equally important; 3 = moderately important; 5 = strongly important; 7 = very strongly important; and 9: extremely important. The sensitivity was checked by calculating the consistency index for each expert for the ranking score of the criteria. An index less than or equal to 0.10 indicates that the experts are consistent in their pairwise comparisons. Inconsistencies in an expert's judgments can be resolved by the expert or removed from the analysis if the inconsistencies are not resolved.^76^

### Multi‐criteria decision analysis

Multi‐criteria decision analysis is used to achieve a goal (a level of success) by evaluating different alternatives based on multiple criteria.^77^ The MCDA algorithm has been used when comparing electricity generation technologies, packaging materials,^47^, ^59^, ^78^ or modern construction methods.^34^

TOPSIS identifies the optimal solution by evaluating similarity or proximity to the best solution, including uncertainty, whereas SAW aggregates weighted evaluations.^79^ TOPSIS evaluates alternatives (products) after calculating the Euclidean distances of an alternative to the ideal (for maximized criteria) and anti‐ideal (for minimized criteria) solutions, as depicted in Supporting Information, Fig. S1.

In this study, as there was no product that was best (or dominant) on all sustainability criteria, a decision needed to be made to find non‐dominant (Pareto optimal) alternatives in the multi‐dimensional space. The selection and ranking problem is solved by structuring the problem in the form of a multi‐criteria decision matrix. There are seven subcategories of influence, which implies a seven‐dimensional space. Since the social inventory in the SHDB is expressed as medium risk hours, there should be minimized, while product‐specific criteria should be maximized.

## RESULTS AND DISCUSSION

### The roadmap to calculate the social sustainability of food products

**Figure 3:**
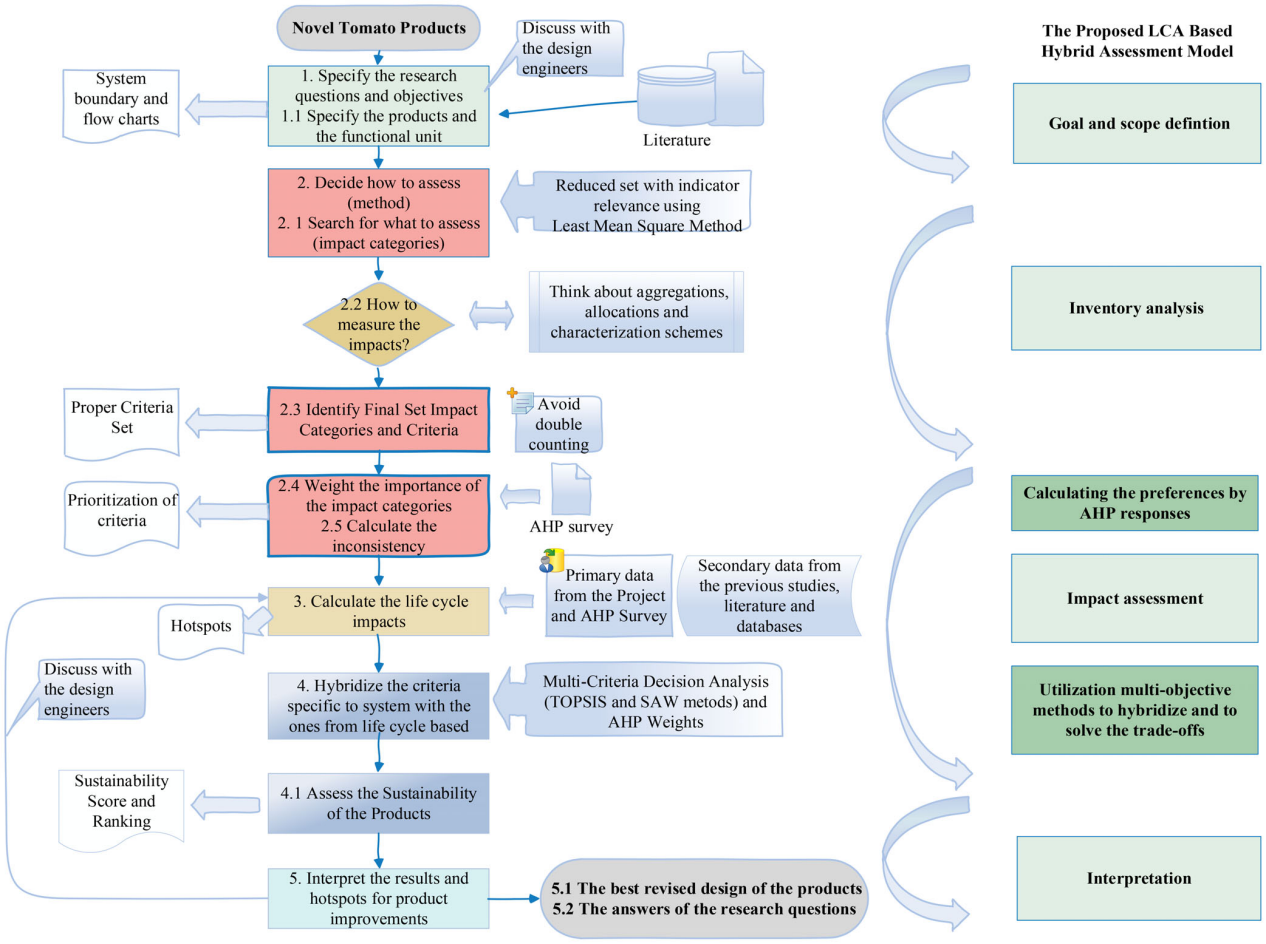
Roadmap for the methodological steps.

Appropriate criteria, subcriteria, and related indicators were identified to assess the social impacts of dried tomato products in this study. One can combine different decision‐making methods to achieve comprehensive results with life cycle assessment of social sustainability. As a result, the proposed road map consisted of LCA stages^80^ (objective and scope definition, inventory acquisition, impact assessment process, and interpretation) with a combination of two additional steps. First, interviews with a set of experts were conducted on an AHP survey to establish the prioritization of impact categories (criteria). Second, product‐specific (micro‐level) criteria were integrated with LCA‐based social indicators using MCDA methods. When studying the social sustainability of a specific product life cycle, we demostrated that the product specifications should be combined with global social issues to achieve a comprehensive assessment. The roadmap in Fig. 3 can be followed to evaluate the social LCA sustainability of other food‐product life cycles.

### Criteria prioritization in the proposed sustainability study

The AHP method uses scales called ‘folds’ in pairwise comparision, where each criterion's importance is rated. For example, if criterion B is three times more important than criterion A, importance is assigned a value 3). As a result, the geometric mean of the expert's responses is recommended to calculate the weights in a consistent way.^76^ Thus, the answers of 11 experts were aggregated using geometric means. For each impact subcategory, equal weighting of criteria is assumed. The final weight (W) for each impact criterion was determined by multiplying the weight of the impact category by the subcategory weight (all category criteria in a subcategory received equal weight). For instance, if an impact category is weighted at 26.8% (for employer), a subcategory 63% (for employment), with four criteria under this subcategory (employment) as detailed in Table 4, the weight for each criterion was calculated by multiplying 26.8% by 63% and then dividing by four (0.25), resulting a final weight of 4.2%.

**Table 4:** Weights of the impact categories, subcategories, and criteria with goals in the social assessment model

Impact category	Weight (%)	Impact subcategory	Weight (%)	Impact criteria	Best	Weight (%)
Worker/employer	26.8	Employment	63	Unemployment	Min.	4.2
				Discrimination	Min.	4.2
				Migrant labor	Min.	4.2
				Child labor	Min.	4.2
		Labor rights	37	Forced labor	Min.	2.5
				Excessive working time	Min.	2.5
				Freedom of association	Min.	2.5
				Labor laws	Min.	2.5
Consumer	35.5	Sensory satisfaction	28	Sensory analysis	Max.	10
		Nutritional quality	72	Total phenolic content (TPC + flavonoid content	Max.	8.5
				DPPH content	Max.	8.5
				Protein content	Max.	8.5
Society	37.7	Benefits to research and innovation	19	Research and innovation capacity	Max.	7.1
		infrastructural improvements	46	Access to drinking water	Min.	4.4
				Access to sanitation	Min.	4.4
				Children out of school	Min.	4.4
				Access to hospital beds	Min.	4.4
		Human rights	35	Gender equity	Min.	3.3
				Indigenous rights	Min.	3.3
				High conflicts	Min.	3.3
				Corruption	Min.	3.3

Based on the weight results in Table 4, stakeholder prioritization was calculated as follows: IC1 for worker/employer is 26.8%, IC2 for customer is 35.5% and IC3 for society is 37.7% (*λ*max = 3.08, IR (index random consistency) = 0.53 and consistency ratio = 0.08, which should be <0.01). Among the impact criteria, the goal of MCDA should be to minimize when the criterion is risky and to maximize when the criterion is beneficial.

**Figure 4:**
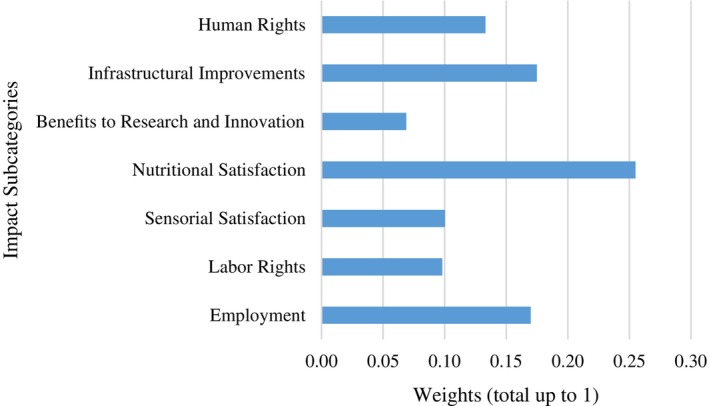
Weights of social impact subcategories.

As shown in Fig. 4, nutritional quality, which is not a criterion in the LCA model of SHDB, was validated at this highest level of importance for inclusion in our social modeling study. The criteria with the second highest importance are employment and infrastructural improvements, with a value of 17%. Employment was a criterion under employer/labor, and infrastructural improvements was a criterion under society stakeholder.

The other proposed criterion, sensory benefits, has the same importance (10%) as labor rights, which are already included in the SHDB. Although sensory impact is not included in the social LCA model of SHDB, the prioritization once again reveals the importance of this criterion. Under the society heading, the criterion of developing research and innovation capacity, which is not part of the SHDV model but was included in this study, is regarded as the least important. However, it is still significant at 7% (*λ*max = 3.0, IR (index random consistency) = 0.53 and consistency ratio = 0 (should be <0.01)).

### Social impacts of novel tomato products

**Figure 5:**
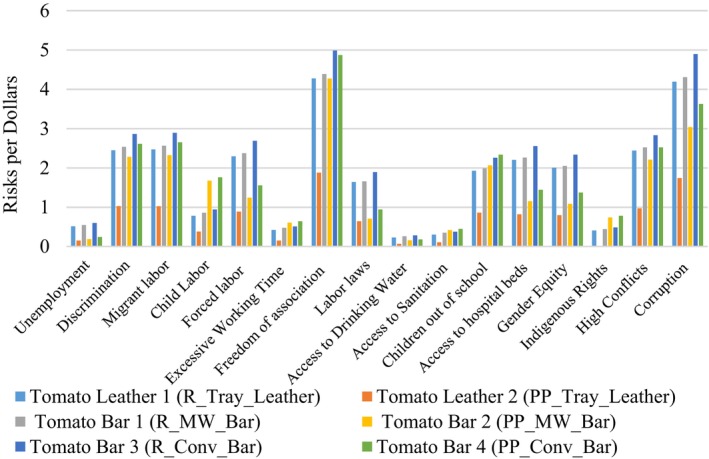
Social impacts of the novel tomato products according to the impact criteria.

This step analyzes social LCA outputs to identify and address high‐risk activities without specific weights. It highlights key risks within each activity of the product life cycle, and regional risks, enabling adjustments to the supply chain, materials, or stakeholders to avoid high‐risk areas. Figure 5 shows notable social risks in freedom of association (under the category of labor rights) and corruption (under the category of human rights). Product life cycles have minimal impact on unemployment, access to drinking water, sanitation, indigenous rights, or excessive working hours, compared to impact on discrimination, migrant labor, children out of school, and access to hospital beds.

**Figure 6:**
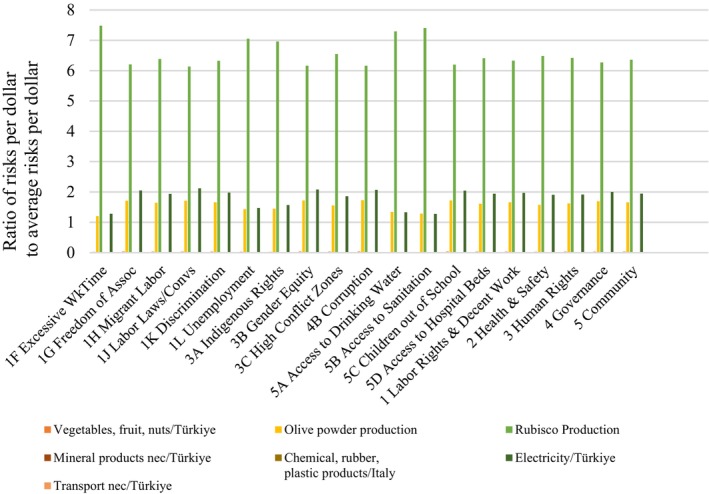
Social footprint of tomato leather product‐1 (Rubisco_tray dryer_leather) by sector and region.

**Figure 7:**
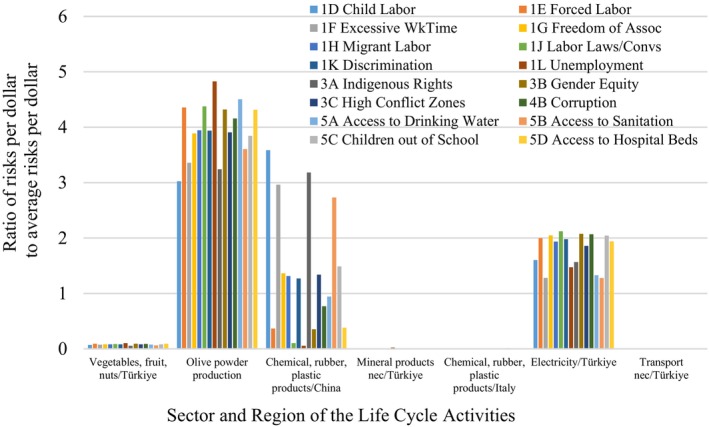
Social footprint of tomato leather product‐2 (pea protein_tray dryer_leather) by sector and region.

Figures 6 and 7 provide the social hotspots (risk areas) due to the materials and processes according to the sector and region given in Supporting Information, Table S5.

It reveals that Rubisco protein contributes more social burdens than the processing method itself. The hotspots include olive powder production (yellow), Rubisco protein production (green), and electricity consumption (dark brown) associated with these processes. When pea protein source is used, the social risks are reduced. In this case, pea protein also has some social risks, although they have less of an impact than does energy, as shown in Fig. 7. Olive powder as a raw material for bar and leather products also has social burdens with regard to sustainability. In this step, the regional risks can be observed.

Supporting Information, Figures S2 and S3 highlight the significant social risks associated with Rubisco (gray) and olive powder (green) components. Their life cycles, shown in Supporting Information, Figs S4 and S5, play crucial roles in product sustainability. The use of a freeze dryer for both components increases social hotspots. The pectinase enzyme also poses social risks for Rubisco production. Supporting Information, Fig. S6 shows the life cycle of tomato snack bar product‐3 with Rubisco protein and a conventional dryer, indicating increased social risks due to the energy‐intensive process. Key social hotspots include Rubisco protein and olive powder production.

Detailed analysis of hotspots for tomato bar product‐4 with pea protein (Supporting Information, Fig. S7) shows significant social risks in child labor, excessive working time, and access to sanitation from the pea protein source, even more than from electricity use, the conventional dryer process, and olive powder production.

Supporting Information, Figure S8 summarizes the social risks for all novel tomato products. The most prominent risks are in freedom of association (labor rights) and corruption (human rights). The smallest risks are in unemployment, access to drinking water, sanitation, and indigenous rights, with minimal risk from excessive working time.

**Figure 8:**
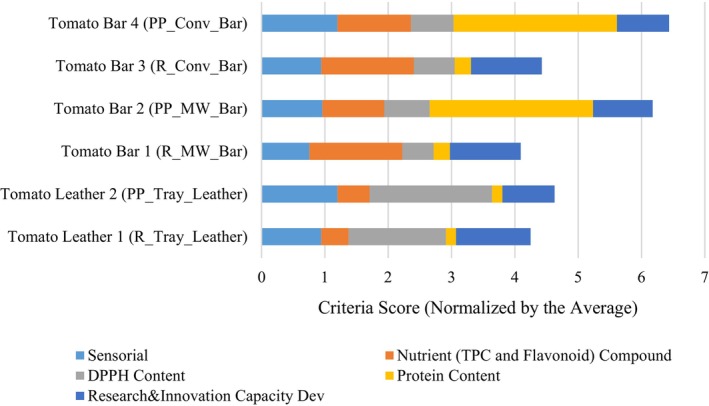
The product‐specific beneficial criteria after normalization with the average of each criterion.

The proposed model includes attributes related to product benefits. Figure 8 shows micro‐level social benefits. Tomato bar product‐2 with pea protein and MW vacuum dryer scores highest in sustainability among bar products. Leather products have higher DPPH antioxidant capacity. Increased protein in the bars boosts protein content. Bar products also have high phenolic content, which is beneficial for a healthy diet.

### Sustainability scores and ranking of dried tomato products

This section prioritizes criteria by integrating risks and benefits using MCDA and AHP preferences, calculating sustainability scores to rank products.

**Figure 9:**
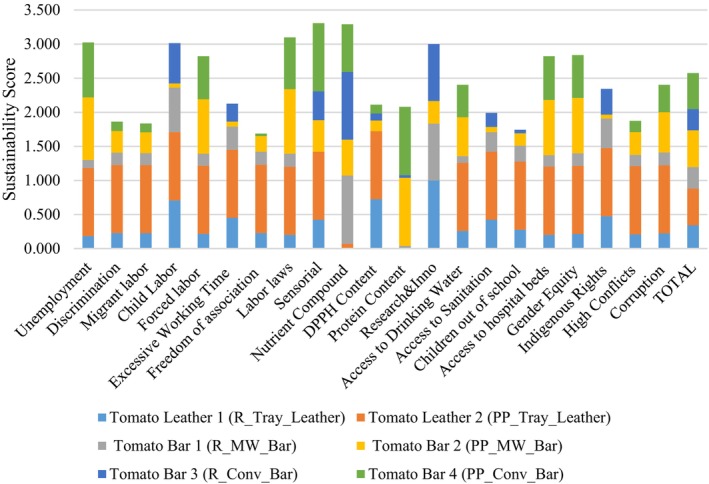
The social sustainability score of each product based on criterion by TOPSIS after normalization.

Social risks in hotspots can vary based on AHP importance. Figure 9 shows sustainability scores for each impact criterion. Supporting Information, Tables S6 and S7 present the decision matrices, and Supporting Information, Table S8 shows rankings with TOPSIS and SAW. Unemployment, labor laws, sensory results, nutritional utility, research and innovation capacity, gender equality, child labor, and forced labor were associated with higher sustainability, whereas discrimination, migrant labor, and labor laws were associated with lower sustainability. The best products were tomato leather‐2 with pea protein and tomato snack bar‐2 with pea protein, both using microwave vacuum drying (Supporting Information, Fig. S9).

The findings show that the sensory analysis and nutrient content embedded in the social aspects at the global level changed the sustainability score and sustainability ranking of the products. The results were compared with the SHDB's social LCA method. As the SHDB impact categories indicate risks, lower values are better (Supporting Information, Fig. S10). Supporting Information, Table S9 presents impact category results from both the proposed model and SHDB. The best products, which are tomato leather‐2 (PP, tray) and tomato bar‐2 (PP, MW) align with the model's findings. Differences in subsequent rankings are due to product‐specific criteria like nutrient content, sensory results, and innovation capacity considered in our model, which integrates both product‐specific criteria and macro‐level data from SHDB.

### Improvement measures and actions

Strategies for minimizing hotspots in the social sustainability of the novel tomato products are summarized in Table 5.

**Table 5:** Negative aspects of the designed food system

Hotspots	Identified critical aspects	Improvement
Rubisco production	Freeze dryer is energy intense	Other alternatives such as hybrid methods may be tried. Other plant‐based proteins can be tried.
Rubisco production	Pectinase enzyme	Purchasing stakeholder may shift to regions with lower global level impacts. Product recipes may be altered to avoid using of enzymes.
Pea protein	Protein content	It increases the product's social risks more than the energy load of the dryer as seen in the case of tomato bar product‐4. The purchasing origin of stakeholder can be changed. Other protein alternatives can be tried.
Olive powder production	Freeze dryer	Drying technology with renewable energies or hybrid drying technologies with MW vacuum may be tried.

The beneficial aspects for maximizing product social sustainability are listed in Table 6, which also summarizes the critical points identified in the literature.

**Table 6:** Beneficial aspects of designed food system

Life cycle activity	Positive aspects (benefits)	Critical points
Raw materials	Nutritional quality	The protein content was enriched in the products. The antioxidant activity was increased with the addition of olive powder. However, Rubisco and olive powder life cycles are also a concern to be improved.
	DPPH content	Lycopene content may also be analyzed and modeled in later activities.
	Pea protein	This increases the product social risks more than energy load dryer as in the case of tomato bar product‐4. The raw material purchasing stakeholder can be changed. Other protein alternatives can be tried.
Drying	Total Phenolic Conten	Microwave vacuum dryer protects total henolic content
	Sensory property	The energy results for a conventional dryer are not as bad as for a tray dryer. It also increased the sensory utility of the products.

## CONCLUSION

This study used life cycle assessment (LCA) to evaluate the social sustainability of novel tomato products. The roadmap offers decision‐makers flexibility in the selection and integration of criteria using MCDA. Unlike the Social Hotspots Database (SHDB), the approach taken in this study includes product‐specific criteria for a more precise social potential estimate. This model also allows for excluding certain criteria to avoid double counting in sustainability assessments with more than one dimension. Key criteria identified through AHP responses include nutrient content, sensory results, and research and innovation capacity, with a particular emphasis on nutritional sustainability, which is a crucial sustainable development goal.

Our results show that regional risks can be mitigated by altering supply chains, materials, and stakeholders. High‐risk hotspots can be reduced by selecting protein sources and origins carefully, achieving sustainability improvements by modifying ingredients or purchasing countries.

Future research should aim to reduce waste through mitigation procedures at each stage, to achieve zero‐waste supply chains in food systems. Diversifying weighting methods, exploring other MCDA solutions for uncertain data, and performing different sensitivity analyses are recommended. The findings provide a framework for food companies to design and evaluate product sustainability.^81^

## AUTHOR CONTRIBUTIONS

Dilber Ayhan conceived the study, conceptualized the LCA methodology, conducted the data analysis and sustainability assessments and wrote the manuscript. Mecit Halil Oztop provided the research funding and coordinated the funded project. Muhammed Rasim Gul contributed to the production of the novel products. Izzet Ari contributed to the structure of the paper. Mecit Halil Oztop and Hami Alpas supervised. All authors reviewed, provided feedback, and approved the final manuscript.

## CONFLICT OF INTEREST

The authors declare no conflicts of interest.

## ETHICS STATEMENT

The ethics committee of Middle East Technical University has no objections to the publication of this work.

## Supporting information


**Data S1:** Supporting Information

## Data Availability

The data that supports the findings of this study are available in the supplementary material of this article.
