# Supplementary material for: Social life cycle sustainability assessment of dried tomato products based on material and process selection through multi‐criteria decision making
Source: J Sci Food Agric. 2024 Oct 26;105(3):1978–92. doi: 10.1002/jsfa.13974 (PMC11726593; doi:10.1002/jsfa.13974)
Supplement: Supplementary file 1 — Data S1: Supporting Information [file JSFA-105-1978-s001.docx]

**Supporting Information**

**Social Life Cycle Sustainability of Dried Tomato Products Based on Material and Process Selection Through Multi-criteria Decision Making**

Dilber Ayhan, Francisco Astorga Mendoza, Muhammed Rasim Gul, Izzet Ari, Hami Alpas, Mecit Halil Oztop*

- Dilber Ayhan, Scientific and Technological Research Council of Türkiye (TUBITAK), Ankara, Türkiye, [dilber@ayhan@tubitak.gov.tr](mailto:dilber@ayhan@tubitak.gov.tr)
- Francisco Astorga Mendoza, Lomartov Applied Innovation Engineering, Valencia, Spain, [fjastorga@gmail.com](mailto:fjastorga@gmail.com)
- Muhammed Rasim Gul, Food Engineering, Middle East Technical University, Ankara, Türkiye, [rasim@metu.edu.tr](mailto:rasim@metu.edu.tr)
- Izzet Ari, Graduate School of Social Sciences, Social Sciences University of Ankara, Ankara, Türkiye. [izzet.ari@asbu.edu.tr](mailto:izzet.ari@asbu.edu.tr)
- Hami Alpas, Food Engineering, Middle East Technical University. Earth System Science, Graduate School of Natural and Applied Sciences, Middle East Technical University, Ankara, Türkiye, [imah@metu.edu.tr](mailto:imah@metu.edu.tr)
- Mecit Halil Oztop, Food Engineering, Middle East Technical University, Ankara, Türkiye, [mecit@metu.edu.tr](mailto:mecit@metu.edu.tr)

***Corresponding Author:** Mecit Halil Oztop, e-mail: [mecit@metu.edu.tr](mailto:mecit@metu.edu.tr) OR: 0000-0001-6414-8942

**Figures S1-S10**

**Tables S1-S9**

**FIGURES**

*Figure S1:* The steps of the TOPSIS algorithm (Sánchez-Lozano et al., 2013)

*Figure S2:* Social footprints of tomato bars with rubisco protein and MW vacuum dryer in terms of sector and origin

*Figure S3*: Social footprints of tomato bar with pea protein and MW vacuum dryer in terms of sector and origin.

*Figure S4*: Social footprints of the rubisco protein production in terms of sector and origin

*Figure S5:* Social footprints of the olive powder production in terms of sector and origin. The greatest risks associated with freeze-drying of olive powder are corruption and freedom of association.

*Figure S6:* Tomato bar with rubisco and conventional dryer social footprints in terms of sector and origin

*Figure S7:* Social footprints of tomato bar with pea protein and conventional dryer in terms of sector and origin. The food product nec, which refers to pea protein from China, has the highest risks in terms of child labor, access to sanitation and indigenous rights. Olive powder production in Türkiye also has some drawbacks in terms of unemployment and labor laws.

*Figure S8*: Social risks of six novel tomato products according to impact criteria

*Figure S9:* Social sustainability with TOPSIS with AHP weighting. The most sustainable product among the bars is tomato bar with pea protein and MW vacuum drying. Compared with the Rubisco product, pea protein is more sustainable for tomato leather.

Figure S10: Social footprints according to the impact categories of SHDB. This indicates that the risks are opposite to those in Figure 8. Among the bar products, tomato bar with pea protein and MW vacuum drying is the greatest sustainable one.

**TABLES**

Table S1. *Composition of the tomato snack bars (45 grams samples)*

| Ingredients | Amounts (g) |
| --- | --- |
| Tomato juice | 100 |
| *Pea Protein Isolate/Rubisco | 10/1 |
| *Tomato powder | 10/19 |
| Salt | 2 |
| Olive powder | 2 |
| Red Pepper powder | 1 |
| Thyme | 1 |
| Mint | 1 |
| Low Methoxyl Pectin (LMP) | 1 |

*****The protein amount and type differ. If rubisco protein is used, the protein amount is decreased to 1 gram, but the tomato powder amount is increased to 19 grams to compensate for the total weight.

Table S2*: Impact categories of the SHDB (Benoit & Mazijn, 2009; Benoit-Norris & Norris, 2015)*

| **Impact Categories** | Labor rights and decent work | Health and safety | Human rights | Governance | Community infrastructure |
| --- | --- | --- | --- | --- | --- |
| **Subcategories** | *Child labor*  *Forced labor*  *Excessive working time*  *Wage assessment*  *Poverty*  *Migrant labor*  *Freedom of association*  *Unemployment*  *Labor laws*  *Discrimination*  *Social Benefits* | *Injuries and fatalities*  *Toxics and hazards* | *Indigenous rights*  *High conflicts*  *Gender equity*  *Human health issues* | *Legal systems*  *Corruptions* | *Hospital beds*  *Drinking water*  *Sanitation*  *Children out of school*  *Smallholder vs. commercial farms* |

Table S3: *The specifications of the experts in the AHP survey*


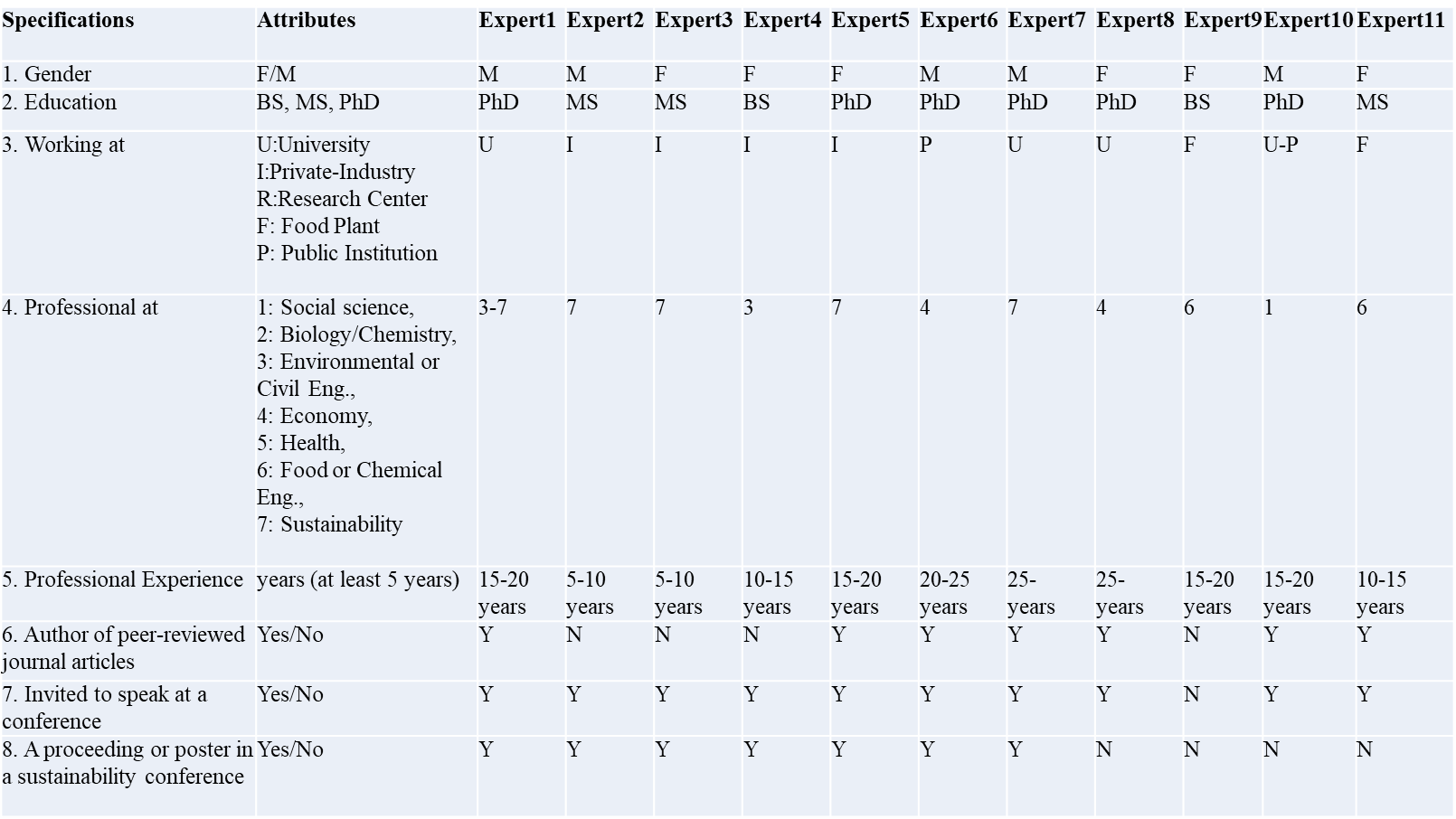


Table S4: *AHP questionnaire summary for social criteria*

| Pairwise Comparisons | | |
| --- | --- | --- |
| “Employer/Worker” | versus | “Consumer” |
| “Employer/Worker” | versus | “Society” |
| “Consumer” | versus | “Society” |
| “Employment” | versus | “Labor Rights” |
| “Sensorial Satisfaction” | versus | “Nutrient Content” |
| “Benefits to Research and Innovation Capacity” | versus | “Infrastructural Improvements” |
| “Benefits to Research and Innovation Capacity” | versus | “Human Rights” |
| “Infrastructural Improvements” | versus | “Human Rights” |

Table S5: *Sector and region analysis for data acquisition from the SHDB*

| **Inventory** | **Selected sector in the SHDB/region** |
| --- | --- |
| Pea Protein Isolate | Food products nec/China |
| Chemical ingredients for Rubisco Protein | Food products nec/Germany |
| Mint, Thyme, Red Pepper | Food products nec/Türkiye |
| Salt | Mineral products nec/Türkiye |
| Sugarcane, sugar beet for Rubisco Protein Production | Sugarcane, sugar beet/Türkiye |
| Green olives for olive powder production | Vegetable oils and fats/Türkiye |
| Tomato and tomato pomace | Vegetables, fruit, nuts/Türkiye |
| Water | Water/Türkiye |
| Chemicals in Rubisco Protein Production | Chemical, rubber, plastic products/Germany |
| Low Methoxyl Pectin | Food products nec/France |
| Pectinase Enzyme | Chemical, rubber, plastic products/Denmark |
| Biodegradable packaging material | Chemical, rubber, plastic products/Italy |
| Processes | Electricity/Türkiye |
| Transportation | Transport nec/Türkiye |

Table S6: *Social Decision Matrix of TOPSIS after Weighting and Normalization*

| **Criteria** | **A1** | **A2** | **A3** | **A4** | **A5** | **A6** |
| --- | --- | --- | --- | --- | --- | --- |
| **C1** | 0.01512 | 0.00000 | 0.01638 | 0.00148 | 0.01854 | 0.00364 |
| **C2** | 0.01036 | 0.00000 | 0.01099 | 0.00914 | 0.01340 | 0.01156 |
| **C3** | 0.01042 | 0.00000 | 0.01111 | 0.00937 | 0.01346 | 0.01172 |
| **C4** | 0.00595 | 0.00000 | 0.00707 | 0.01911 | 0.00833 | 0.02037 |
| **C5** | 0.00723 | 0.00000 | 0.00763 | 0.00183 | 0.00923 | 0.00343 |
| **C6** | 0.00546 | 0.00000 | 0.00660 | 0.00921 | 0.00734 | 0.00995 |
| **C7** | 0.00565 | 0.00000 | 0.00592 | 0.00565 | 0.00733 | 0.00706 |
| **C8** | 0.00745 | 0.00000 | 0.00756 | 0.00047 | 0.00933 | 0.00224 |
| **C9** | -0.01040 | 0.00000 | -0.01804 | -0.00971 | -0.01040 | 0.00000 |
| **C10** | -0.03325 | -0.03085 | 0.00000 | -0.01576 | -0.00019 | -0.01002 |
| **C11** | -0.01220 | 0.00000 | -0.04404 | -0.03715 | -0.03945 | -0.03846 |
| **C12** | -0.05591 | -0.05591 | -0.05370 | 0.00000 | -0.05370 | 0.00000 |
| **C13** | 0.00000 | -0.00981 | -0.00164 | -0.00654 | -0.00164 | -0.00981 |
| **C14** | 0.01358 | 0.00000 | 0.01658 | 0.00784 | 0.01833 | 0.00959 |
| **C15** | 0.00988 | 0.00000 | 0.01228 | 0.01580 | 0.01362 | 0.01714 |
| **C16** | 0.00967 | 0.00000 | 0.01023 | 0.01092 | 0.01266 | 0.01336 |
| **C17** | 0.01330 | 0.00000 | 0.01388 | 0.00318 | 0.01668 | 0.00598 |
| **C18** | 0.00964 | 0.00000 | 0.01001 | 0.00231 | 0.01227 | 0.00458 |
| **C19** | 0.01032 | 0.00000 | 0.01111 | 0.01853 | 0.01221 | 0.01964 |
| **C20** | 0.00857 | 0.00000 | 0.00904 | 0.00723 | 0.01084 | 0.00903 |
| **C21** | 0.00882 | 0.00000 | 0.00923 | 0.00466 | 0.01135 | 0.00679 |
| **S*** | 0.07772 | 0.06460 | 0.08379 | 0.05734 | 0.08432 | 0.06062 |
| **S^-^** | 0.04053 | 0.07473 | 0.03898 | 0.06694 | 0.03826 | 0.06788 |
| **Social Sus. Score** | 0.34272 | 0.53632 | 0.31750 | 0.53862 | 0.31210 | 0.52825 |

Table S7: *Social Decision Matrix of SAW after Weighting and Normalization*

| **Criteria** | **A1** | **A2** | **A3** | **A4** | **A5** | **A6** |
| --- | --- | --- | --- | --- | --- | --- |
| **C1** | 0.0126 | 0.0425 | 0.0119 | 0.0345 | 0.0109 | 0.0270 |
| **C2** | 0.0179 | 0.0425 | 0.0173 | 0.0192 | 0.0153 | 0.0168 |
| **C3** | 0.0177 | 0.0425 | 0.0170 | 0.0188 | 0.0151 | 0.0165 |
| **C4** | 0.0206 | 0.0425 | 0.0188 | 0.0096 | 0.0171 | 0.0092 |
| **C5** | 0.0095 | 0.0245 | 0.0092 | 0.0175 | 0.0081 | 0.0140 |
| **C6** | 0.0088 | 0.0245 | 0.0077 | 0.0061 | 0.0072 | 0.0057 |
| **C7** | 0.0108 | 0.0245 | 0.0105 | 0.0108 | 0.0092 | 0.0095 |
| **C8** | 0.0096 | 0.0245 | 0.0095 | 0.0223 | 0.0083 | 0.0167 |
| **C9** | 0.0788 | 0.1003 | 0.0631 | 0.0803 | 0.0788 | 0.1003 |
| **C10** | 0.0249 | 0.0293 | 0.0850 | 0.0565 | 0.0846 | 0.0669 |
| **C11** | 0.0674 | 0.0850 | 0.0217 | 0.0316 | 0.0283 | 0.0297 |
| **C12** | 0.0054 | 0.0054 | 0.0085 | 0.0850 | 0.0085 | 0.0850 |
| **C13** | 0.0688 | 0.0482 | 0.0654 | 0.0550 | 0.0654 | 0.0482 |
| **C14** | 0.0130 | 0.0437 | 0.0113 | 0.0186 | 0.0105 | 0.0164 |
| **C15** | 0.0156 | 0.0437 | 0.0135 | 0.0113 | 0.0125 | 0.0106 |
| **C16** | 0.0195 | 0.0437 | 0.0189 | 0.0182 | 0.0167 | 0.0161 |
| **C17** | 0.0163 | 0.0437 | 0.0159 | 0.0312 | 0.0141 | 0.0249 |
| **C18** | 0.0132 | 0.0333 | 0.0129 | 0.0244 | 0.0114 | 0.0194 |
| **C19** | 0.0000 | 0.0000 | 0.0000 | 0.0000 | 0.0000 | 0.0000 |
| **C20** | 0.0133 | 0.0333 | 0.0129 | 0.0146 | 0.0114 | 0.0129 |
| **C21** | 0.0138 | 0.0333 | 0.0135 | 0.0191 | 0.0118 | 0.0160 |
| **Social Sustainability Score** | 0.4576 | 0.8108 | 0.4443 | 0.5845 | 0.4452 | 0.5616 |

Table S8: *The Social Sustainability Scores By the TOPSIS and SAW Methods after Normalization and Multiplied by AHP Weights*

| Products | TOPSIS Sustainability Score | Ranking | SAW  Sustainability Score | Ranking |
| --- | --- | --- | --- | --- |
| Tomato Leather 1 (R_Tray_Leather) | 0.343 | 4 | 0.458 | 4 |
| Tomato Leather 2 (PP_Tray_Leather) | 0.536 | 2 | 0.811 | 1 |
| Tomato Bar 1  (R_MW_Bar) | 0.318 | 5-6 | 0.444 | 5-6 |
| Tomato Bar 2  (PP_MW_Bar) | 0.539 | 1 | 0.585 | 2 |
| Tomato Bar 3  (R_Conv_Bar) | 0.312 | 5-6 | 0.445 | 5-6 |
| Tomato Bar 4  (PP_Conv_Bar) | 0.528 | 3 | 0.562 | 3 |

Table S9: *Comparison of the Results of the Proposed Model with the Results of the SHDB Impact Categories*

| Method | Subcategories | Products | | | | | |
| --- | --- | --- | --- | --- | --- | --- | --- |
|  |  | A1 | A2 | A3 | A4 | A5 | A6 |
| Sustainability Scores of the Proposed Model  (Better in Positive Direction) | Employment | 1.345 | 4.000 | 1.125 | 1.604 | 0.591 | 1.070 |
|  | Labor Rights | 1.098 | 4.000 | 0.892 | 2.055 | 0.262 | 1.425 |
|  | Sensorial Satisfaction | 0.423 | 1.000 | 0.000 | 0.462 | 0.423 | 1.000 |
|  | Nutritional Satisfaction | 0.723 | 1.072 | 1.039 | 1.682 | 1.138 | 1.825 |
|  | Benefits to Research and Innovation | 1.000 | 0.000 | 0.833 | 0.333 | 0.833 | 0.000 |
|  | Infrastructural Improvements | 1.162 | 4.000 | 0.782 | 1.642 | 0.258 | 1.118 |
|  | Human Rights | 1.122 | 4.000 | 0.972 | 1.790 | 0.378 | 1.196 |
| Risks Given By SHDB Categories  (Better in Negative Direction) | Labor Rights & Decent Work | 34.9988 | 14.7382 | 36.2917 | 32.8652 | 40.9840 | 37.5576 |
|  | Health & Safety | 40.6375 | 16.4299 | 41.9604 | 36.8110 | 47.2346 | 42.0853 |
|  | Human Rights | 22.5853 | 9.1991 | 23.4504 | 19.9945 | 26.3988 | 22.9429 |
|  | Governance | 49.5631 | 21.1455 | 51.3143 | 46.3235 | 58.0714 | 53.0806 |
|  | Community | 26.5549 | 11.0795 | 27.6643 | 24.7981 | 31.1839 | 28.3176 |
